# Intraoperative renal near-infrared spectroscopy indicates developing acute kidney injury in infants undergoing cardiac surgery with cardiopulmonary bypass: a case–control study

**DOI:** 10.1186/s13054-015-0760-9

**Published:** 2015-01-29

**Authors:** Bettina Ruf, Vittorio Bonelli, Gunter Balling, Jürgen Hörer, Nicole Nagdyman, Siegmund Lorenz Braun, Peter Ewert, Karl Reiter

**Affiliations:** Department of Pediatric Cardiology and Congenital Heart Disease, German Heart Centre Munich, Technical University, Lazarettstr. 36, 80636 Munich, Germany; Department of Anaesthesiology, German Heart Centre Munich, Technical University, Lazarettstr. 36, 80636 Munich, Germany; Department of Thoracic and Cardiovascular Surgery, German Heart Centre Munich, Technical University, Lazarettstr. 36, 80636 Munich, Germany; Institute of Laboratory Medicine, German Heart Centre Munich, Technical University, Lazarettstr. 36, 80636 Munich, Germany; Department of Pediatric Intensive Care, University Children`s Hospital, von Haunersche Children hospital, Ludwigs-Maximilian University, Lindwurmstr. 4, 80337 Munich, Germany

## Abstract

**Introduction:**

Acute kidney injury (AKI) is a frequent complication after cardiac surgery with cardiopulmonary bypass in infants. Renal near-infrared spectroscopy (NIRS) is used to evaluate regional oximetry in a non-invasive continuous real-time fashion, and reflects tissue perfusion. The aim of this study was to evaluate the relationship between renal oximetry and development of AKI in the operative and post-operative setting in infants undergoing cardiopulmonary bypass surgery.

**Methods:**

In this prospective study, we enrolled 59 infants undergoing cardiopulmonary bypass surgery for congenital heart disease for univentricular (*n* = 26) or biventricular (*n* = 33) repair. Renal NIRS was continuously measured intraoperatively and for at least 24 hours postoperatively and analysed for the intraoperative and first 12 hours, first 24 hours and first 48 hours postoperatively. The renal oximetry values were correlated with the paediatric risk, injury, failure, loss, end (pRIFLE) classification for AKI, renal biomarkers and the postoperative course.

**Results:**

Twenty-eight (48%) infants developed AKI based on pRIFLE classification. Already during intraoperative renal oximetry and further in the first 12 hours, 24 hours and 48 hours postoperatively, significantly lower renal oximetry values in AKI patients compared with patients with normal renal function were recorded (*P* < 0.05). Of the 28 patients who developed AKI, 3 (11%) needed renal replacement therapy and 2 (7%) died. In the non-AKI group, no deaths occurred. Infants with decreased renal oximetry values developed significantly higher lactate levels 24 hours after surgery. Cystatin C was a late parameter of AKI, and neutrophil gelatinase-associated lipocalin values were not correlated with AKI occurrence.

**Conclusion:**

Our results suggest that prolonged low renal oximetry values during cardiac surgery correlate with the development of AKI and may be superior to conventional biochemical markers. Renal NIRS might be a promising non-invasive tool of multimodal monitoring of kidney function and developing AKI in infants undergoing cardiac surgery with cardiopulmonary bypass.

## Introduction

Acute kidney injury (AKI) is a frequent complication after cardiac surgery with cardiopulmonary bypass (CPB) in infants. In a recent multicentre study including 311 paediatric patients after on-pump cardiac surgery, 42% developed AKI postoperatively [1]. Especially patients younger than 2 years showed an increased risk for AKI [1]. AKI after CPB surgery is associated with longer time on mechanical ventilation and intensive care unit (ICU) stay [1-5] and possibly increased mortality [3,4].

Criteria for the definition of paediatric AKI have been defined by the paediatric modification of the risk, injury, failure, loss, end (pRIFLE) classification. More recently, these criteria were refined by the Acute Kidney Injury Network, but basically they rely on increases in serum creatinine or decreases in creatinine clearance [6]. These parameters reach their maximum in most (78%) of the cases only after 48 hours [1]. Therefore, much effort has been spent on the identification of reliable biomarkers for early detection of AKI (for example, neutrophil gelatinase-associated lipocalin (NGAL), interleukin-18, kidney injury molecule-1 (KIM-1) [7]); however, predictive values vary considerably between studies, and no single substance has been shown to have consistent sensitivity [8]. Most promising in this regard is NGAL, for which, in a recent study in paediatric post–cardiac surgery patients, area under the receiver operating characteristic curve (AUROC) values ranging from 0.68 to 0.92 within the first 15 hours after surgery were found [9].

Near-infrared spectroscopy (NIRS) is a non-invasive, continuous method of evaluating real-time regional oximetry (rSO_2_). The technology is based on the different absorption of near-infrared wavelengths by oxygenated and deoxygenated haemoglobin. The transmitter and the receiving optodes are placed ipsilateral in the sensor. This design capitalizes on the fact that photons transmitted through a sphere will traverse an elliptical path in which the mean depth of penetration is proportional to the transmitter and receiver optode separation [10].

Several studies have shown a moderate correlation between cerebral and somatic NIRS monitoring and markers of cardiac output such as lactate or systemic venous oxygen saturation (ScvO_2_) and regional tissue perfusion in a variety of clinical settings [10-13]. However, NIRS measurements in different organs have shown variable correlations to systemic oxygenation parameters [11,14] or no reliable correlation at all [15].

In a recent study, a correlation was demonstrated between urinary biomarkers and low renal NIRS saturations and a compound outcome, including the need for renal replacement therapy in infants after cardiac surgery [16]. Importantly, no correlation with AKI occurrence was noted.

In our present study, we hypothesized that early and extended continuous renal NIRS recordings starting intraoperatively and continued for at least 24 hours postoperatively would correlate with renal injury measured by biomarkers and pRIFLE stage. We applied a modified renal NIRS threshold to account for low saturations in infants with cyanotic heart lesions and calculated a modified NIRS score that included magnitude and duration of renal desaturations.

## Methods

### Patients and study design

In this prospective study, we consecutively enrolled infants under 12 months of age who underwent cardiac surgery on CPB after written informed consent was obtained from their parents. Infants with preexisting renal disease, sepsis or multiple organ dysfunction were excluded. Ethical approval for the study protocol was given by the research ethics committee at German Heart Centre Munich, Technical University of Munich, Germany.

### Near-infrared spectroscopy

We measured cerebral and renal oximetry with the INVOS monitor (Covidien/Somanetics, Dublin, Ireland) intraoperatively and 24 to 48 hours postoperatively. The NIRS sensor was placed after intubation before starting the operation on the left or right forehead for cerebral oximetry and on the back left or right of the spine at the level of T10-L2 for renal oximetry. Because of the importance of a small skin organ distance, we included only infants younger than 12 month of age and below 10 kg body weight. The rSO_2_ was recorded continuously every 2 seconds after intubation, during CPB and until the end of the surgical procedure. These values represent the intraoperative NIRS measurements. After admission to the ICU, the renal and cerebral oximetry was restarted and recorded continuously for at least 24 hours in each patient. Renal oximetry measurements were continued until extubation or for a maximum of 48 hours postoperatively. The measurements over the first 24 hours postoperatively were calculated for the first 12 hours and the whole first 24-hour period. Values obtained during the 24 hours following the first 24-hour period were analysed separately. In this study, we focused on renal oximetry; therefore, cerebral NIRS values are not presented.

According to our knowledge, validated critical baselines or thresholds for renal rSO_2_ are lacking and do not discern between cyanotic and acyanotic cardiac lesions. We decided to set two different thresholds. According to the critical baseline of 45% to 55% in cerebral NIRS studies [17-19] and the 10 to 20 points higher somatic than cerebral values in healthy infants [20,21], we set the critical baselines at 65% rSO_2_ for renal NIRS. To account for *a priori* lower NIRS values in patients with cyanotic lesions, we defined a second criterion, which includes relative decreases in regional oxygenation compared with an individually calculated baseline. In each patient, following stabilization after intubation, renal rSO_2_ was measured over 10 minutes and values were averaged to define a baseline rSO_2_ value. The threshold was defined as a 25% rSO_2_ decrease compared with the baseline value.

**Figure 1:**
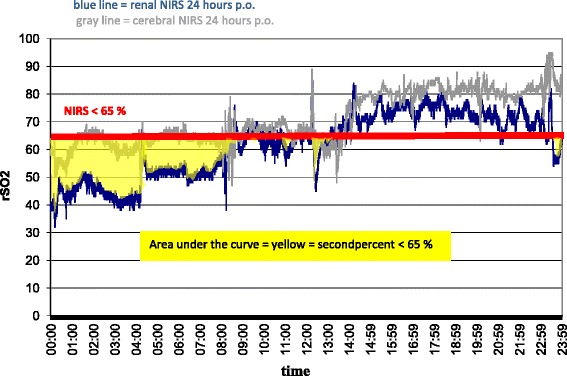
**Example of calculating the area under the curve for regional oxygenation below 65%.** Yellow region represents the second percent with the renal near-infrared spectroscopy (NIRS) below the 65% threshold (red bar). p.o., Postoperatively; rSO_2_, Regional oxygenation.

**Figure 2:**
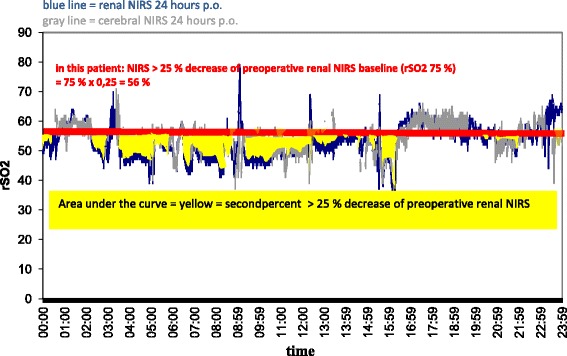
**Example of calculating the area under the curve with the baseline of >25 % decrease of the preoperative individual averaged value of renal near-infrared spectroscopy.** In this case, the area is calculated as 75% × 0.25 = 56%. NIRS, Near-infrared spectroscopy; rSO_2_, Regional oxygenation.

After recording the renal oximetry values intra- and postoperatively, a renal rSO_2_ score was calculated. This score has been established in studies on cerebral NIRS after cardiac surgery in adult patients, but up to now not in infants [17]. It represents an area under the curve (AUC) measurement that reflects both the depth and duration of desaturations below the set thresholds (Figures 1 and 2). We adapted the score for long-term measurements by using minutes instead of seconds in the numerator, which gives the formula: rSO_2_ score = (baseline rSO_2_ − current rSO_2_ (%)) × time (minutes). In this article, the following abbreviation for the two renal NIRS thresholds are used: rNIRS65 = renal rSO_2_ < 65% and rNIRS25 = renal rSO_2_ decrease of 25% compared with a preoperative baseline value.

### Laboratory parameters

Serum creatinine, urea and cystatin C as well as urine volume and urinary creatinine were measured preoperatively, within 1 hour postoperatively and on days 1 to 5 after the surgical procedure.

Creatinine levels in plasma and urine were determined using a kinetic colorimetric assay based on the Jaffé method. For the determination of urea/urea nitrogen in plasma, a kinetic test with urease and glutamate dehydrogenase. For cystatin C measurement, a latex particle-enhanced immunoturbidimetric assay was used. All three tests were done with the Cobas Integra 800 analyser (Roche Diagnostics, Mannheim, Germany).

Urinary NGAL concentrations were measured by performing a solid-phase, enzyme-linked immunosorbent assay based on the sandwich principle (Hycult Biotech, Cambridge, UK). The determination of NGAL in plasma anticoagulated with ethylenediaminetetraacetic acid was performed with a fluorescence immunoassay using the Triage MeterPro (Alere, Cologne, Germany).

### Perioperative course

The following clinical parameters were recorded: operation time, time on bypass, aortic cross-clamp time, circulatory arrest time, time on a respirator, length of stay in the ICU and until hospital discharge. As indirect markers of cardiac output lactate at 6 hours, 12 hours and 24 hours postoperatively, ScvO_2_ and arterial oxygen saturation 2 hours and 24 hours postoperatively were collected. Daily urine output related to body weight and need of renal replacement therapy (peritoneal dialysis or continuous venovenous haemodiafiltration) were noted. The Risk Adjustment for Congenital Heart Surgery (RACHS-1) was used to categorize and compare the severity of the performed cardiac surgery. This instrument gives a score to stratify anatomic diversity into six categories based on type of performed surgery, age at operation and similar in-hospital mortality [22].

According to the pRIFLE classification [6], AKI was defined as a 50% or more increase of serum creatinine. Creatinine was routinely measured preoperatively and in the morning following surgery. Urine volume criteria were not used, owing to variable diuretic use.

### Statistical analysis

We used the *t*-test for independent samples for symmetric data distribution, and we performed a two-sided χ^2^ test for categorical variables and a two-sided Mann–Whitney *U* test for continuous variables in case of an asymmetric data distribution. Data are presented as median and range or as individual values. Correlation analysis was performed with Spearman’s ρ coefficient of correlation for asymmetric data distribution. ROC analysis was used for calculating sensitivity, specificity and positive and negative predictive values. Results were considered statistically significant at *P* < 0.05. For power analysis, we assumed postoperative AKI to occur in 30% of patients. If a clear cutoff value in the NIRS measurements was obtained, we calculated, using standard tables, a sample size of 56 patients to reach a power of 80% and a *P*-value <0.05. The statistical calculations were done using SPSS 21 software (IBM SPSS, Chicago, IL, USA).

## Results

**Figure 3:**
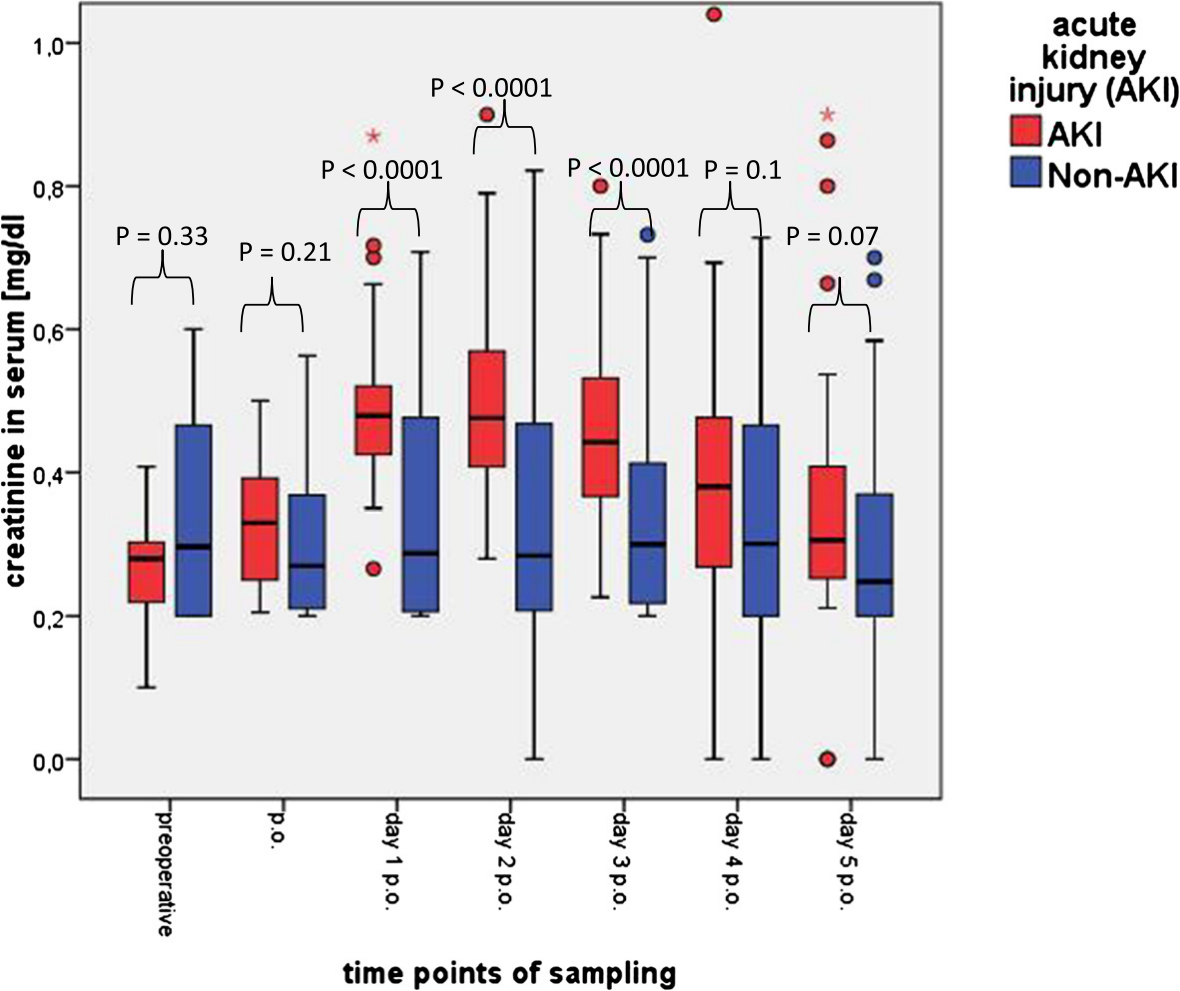
**The pre- and postoperative course of serum creatinine in infants with versus without acute kidney injury who underwent cardiopulmonary bypass surgery is presented as a boxplot.** AKI, Acute kidney injury; p.o., Postoperatively.

From January 2011 to August 2011, we enrolled 59 infants undergoing CPB cardiac surgery because of congenital heart defects. According to the pRIFLE classification scheme [6], we determined that 48% (28 of 59) of the infants developed AKI after CPB. A creatinine increase by 50% of the preoperative baseline value was first seen in the serum creatinine at 24 hours postoperatively; hence, AKI was diagnosed at this time point or later in our cohort. Figure 3 demonstrates the course of serum creatinine levels in infants with versus without AKI.

The infants who developed AKI were significantly younger than the infants who did not. For the demographic data of the two groups, see Table 1.

**Table 1:** **Demographic data of infants with versus without acute kidney injury after undergoing cardiopulmonary bypass surgery** ^**a**^

	**AKI**	**No AKI**	***P*** **-value**
Age at operation (days)	27 (5 to 274)	105 (8 to 305)	0.03
Weight at operation (g)	3,355 (2,445 to 9,100)	4,490 (2,260 to 7,530)	0.1
Height at operation (cm)	52 (44 to 75)	59 (41 to 71)	0.03
Female, *n* (%)	10 (36)	14 (45)	0.8
Male, *n* (%)	18 (64)	17 (55)	
BSA at operation (m^2^)	0.22 (0.17 to 0.41)	0.26 (0.15 to 0.36)	0.06

^a^The demographic data are listed as median and range or individual values. AKI, Acute kidney injury; BSA, Body surface area.

There was no significant difference between the two groups in operation time, bypass time, aortic cross clamp and circulatory arrest time. The infants who developed AKI were significantly more often palliated with an aortopulmonary shunt than infants without AKI. The RACHS-1 was not significantly different between the two groups. The surgical data and RACHS-1 scores are listed in Table 2.

**Table 2:** **Surgery-related data and Risk Adjustment for Congenital Heart Surgery scores of infants with versus without acute kidney injury after undergoing cardiopulmonary bypass surgery** ^**a**^

		**AKI**	**No AKI**	***P*** **-value**
Biventricular correction, *n* (%)		11 (39%)	22 (71%)	0.01
Univentricular palliation: cyanotic, *n* (%)		17 (61%)	9 (29%)	0.01
Shunt, *n* (%)		15 (54%)	3 (10%)	P < 0.01
PCPC, *n* (%)		2 (7%)	6 (19%)	
Operation time (min)		174 (80 to 530)	150 (80 to 555)	0.45
Bypass (min)		75 (30 to 150)	77 (23 to 160)	0.9
Aortic cross-clamp (min)		28 (0 to 83)	47 (0 to 110)	0.07
Circulatory arrest (min)		0 (0 to 53)	0 (0 to 44)	0.12
RACHS-1 score				
	2 and 3, *n* (%)	15 (56)	22 (69)	0.42
	4 to 6, *n* (%)	12 (44)	10 (31)	0.42

^a^The operation data and the Risk Adjustment for Congenital Heart Surgery (RACHS-1) score are listed as median and range or individual values. AKI, Acute kidney injury; PCPC, Partial cavopulmonary connection.

Plasma lactate was significantly higher and ScvO_2_ significantly lower at 24 hours postoperatively in the AKI group versus the non-AKI group (Table 3). Three (11%) of twenty-eight infants with AKI needed renal replacement therapy (one peritoneal dialysis, two continuous venovenous haemodiafiltration) after resuscitation at the end of surgery (one case) and on postoperative days 3 and 4 (two cases). Two (7 %) of twenty-eight infants with AKI died. There was no fatal course in the non-AKI group. Time on mechanical ventilation was significantly longer in infants with AKI compared with infants without AKI. Table 3 presents the clinical data of the postoperative course.

**Table 3:** **Postoperative clinical data of infants with versus without acute kidney injury after undergoing cardiopulmonary bypass surgery** ^**a**^

	**AKI**	**No AKI**	***P*** **-value**
Lactate 6 hr p.o. (μmol/L)	2.2 (0.9 to 17)	1.9 (0.9 to 7.4)	0.32
Lactate 12 hr p.o. (μmol/L)	3 (1.1 to 19)	2.4 (1 to 6)	0.08
Lactate 24 hr p.o. (μmol/L)	2.6 (1.4 to 16)	1.7 (0.9 to 6.5)	0.01
ScvO_2_ 2 hr p.o. (%)	57 (35 to 88)	60 (34 to 87)	0.54
ScvO_2_ 24 hr p.o. (%)	55 (40 to 88)	62 (44 to 89)	0.004
SaO_2_ 2 hr p.o. (%)	87 (70 to 100)	92 (55 to 99)	0.39
SaO_2_ 24 hr p.o. (%)	83 (69 to 100)	93 (63 to 100)	0.76
MAP <50 mmHg >2 hr within 24 hr p.o., *n* (%)	16 (59)	8 (25)	0.008
Norepinephrine ≤0.1 μg/kg/min, *n* (%)	15 (56)	11 (34)	0.03
Norepinephrine >0.1 μg/kg/min, *n* (%)	5 (19)	2 (6)	0.01
Urine output 24 hr p.o. (ml/kg)	4.9 (1.5 to 7.9)	4.6 (2.9 to 8.8)	0.37
Time on respirator (hr)	47 (2 to 624)	22 (2 to 270)	0.03
ICU stay (days)	12 (3 to 35)	10 (4 to 74)	0.06
Hospital stay (days)	28 (7 to 76)	25 (12 to 96)	0.13
RRT, *n* (%)	3 (11)	0 (0)	0.09
Deaths, *n* (%)	2 (7)	0 (0)	0.2

^a^The clinical data of the postoperative course are presented as median and range or individual values. AKI, Acute kidney injury; ICU, Intensive care unit; MAP, Mean arterial pressure; p.o., Postoperatively; RRT, Renal replacement therapy; SaO_2_, Arterial oxygen saturation; ScvO_2_, Systemic venous oxygen saturation.

**Figure 4:**
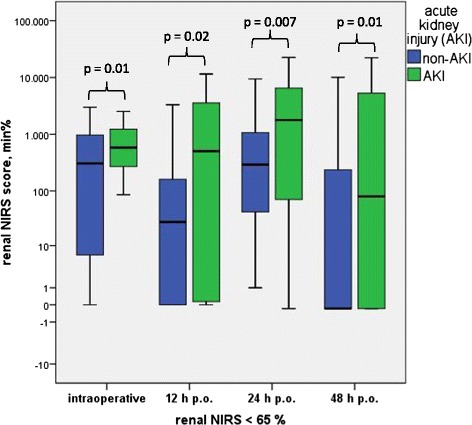
**Renal near-infrared spectroscopy score in minute percent intraoperatively and 12, 24 and 48 hours postoperatively below the set baseline of regional oximetry <65% in infants with versus without acute kidney injury undergoing cardiopulmonary bypass operations.** AKI, Acute kidney injury; NIRS, Near-infrared spectroscopy; p.o., Postoperatively.

**Figure 5:**
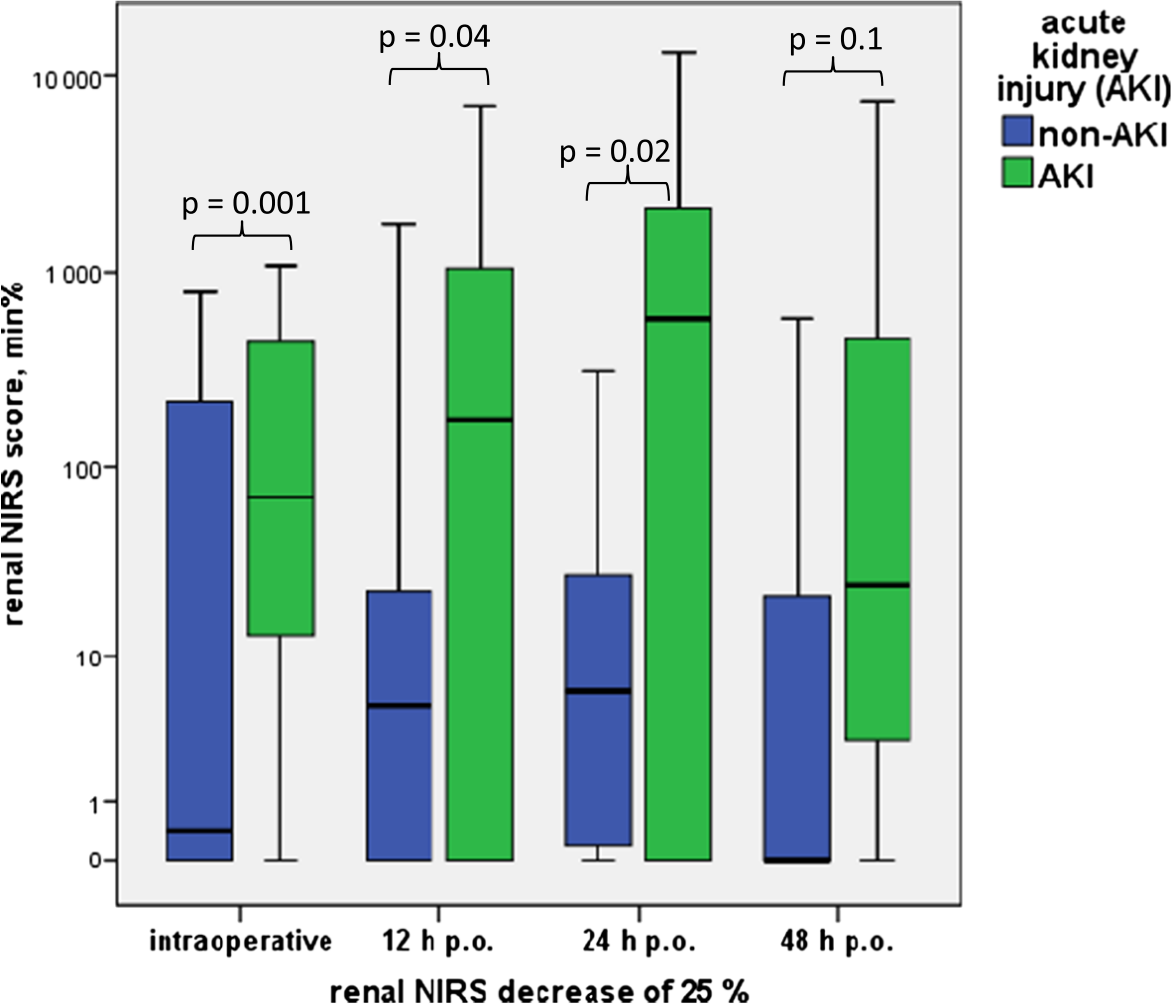
**Renal near-infrared spectroscopy score in minute percent intraoperatively and at 12, 24 and 48 hours postoperatively below the set threshold regional oximetry decrease >25% of the preoperative averaged baseline value in infants with versus without acute kidney injury undergoing cardiopulmonary bypass operations.** AKI, Acute kidney injury; NIRS, Near-infrared spectroscopy; p.o., Postoperatively.

The renal rSO_2_ during the intraoperative course showed significantly higher rSO_2_ scores (and therefore worse oxygenation values) in infants with AKI than in infants without AKI for both baseline definitions. Infants with AKI demonstrated a significantly higher median rNIRS65 score (598 min%; range: 86 to 2,532; *P* = 0.01) compared with infants without AKI (154 min%; range: 0 to 2,997; *P* = 0.001). The median rNIRS25 score was also significantly higher in infants with AKI (131 min%; range: 0 to 1,200) than in infants without AKI (0 min%; range: 0 to 2,263; *P* = 0.001) (Table 4, Figures 4 and 5). Postoperatively, both renal rSO_2_ scores were significantly higher 12 and 24 hours after surgery in the AKI versus the non-AKI group (rNIRS65 score at 12 hours: 694 (0 to 11,534) min% in the AKI vs. 26 (0 to 3,124) min% in the non-AKI group (*P* = 0.02); rNIRS65 at 24 hours: 3,747 (0 to 22,590) min% vs. 267 (1 to 9,417) min% in the non-AKI group; *P* = 0.007) (Table 4, Figures 4 and 5). At 12 hours, infants with AKI showed a rNIRS25 score of 4 (0 to 7,091) min% compared with 0 (0 to 1,772) min% in infants without AKI (*P* = 0.04), and at 24 hours, rNIRS25 scores were 214 (0 to 13,267) min% compared with 4 (0 to 312) min% in the AKI versus non-AKI group, respectively (*P* = 0.02) (Table 4, Figures 4 and 5). Forty-eight hours postoperatively, only the rNIRS65 score was significantly higher in the AKI in comparison with the non-AKI group.

**Table 4:** **Intra- and postoperative renal near-infrared spectroscopy scores of infants with versus without acute kidney injury** ^**a**^

	**AKI**	**No AKI**	***P*** **-value**
Renal NIRS <65% intraoperative (min%)	598 (86 to 2,532)	154 (0 to 2,997)	0.01*
Renal NIRS <65% 12 hr p.o. (min%)	694 (0 to 11,534)	26 (0 to 3,124)	0.02*
Renal NIRS <65% 24 hr p.o. (min%)	3,747 (0 to 22,590)	267 (1 to 9,417)	0.007*
Renal NIRS <65% 48 hr p.o. (min%)	1,113 (0 to 21,740)	150 (0 to 8,153)	0.04*
Renal NIRS ∆25% intraoperative (min%)	131 (0 to 1,200)	0 (0 to 2,263)	0.001*
Renal NIRS ∆25% 12 hr p.o. (min%)	4 (0 to 7,091)	0 (0 to 1,772)	0.04*
Renal NIRS ∆25% 24 hr p.o. (min%)	214 (0 to 13,267)	4 (0 to 312)	0.02*
Renal NIRS ∆25% 48 hr p.o. (min%)	25 (0 to 6,935)	0 (0 to 3,518)	0.1

^a^The renal near-infrared spectroscopy (NIRS) decrease below the baseline of 65% and the decrease of 25% below the individualized preoperative renal NIRS intraoperatively and 12, 24 and 48 hours postoperatively are expressed as median and range. AKI, Acute kidney injury; p.o., Postoperatively. *P < 0.05 indicates statistically significant values.

Renal NIRS values of patients with cyanosis compared with patients without cyanosis independent of AKI showed no significant difference at the given time points. There was a moderate negative correlation between the arterial oxygen saturation 2 hours postoperatively with the rNIRS65 score within 12 hours (*r* = 0.5; *P* = 0.01) and 24 hours (*r* = 0.4; *P* = 0.07) after surgery, but not the postoperative rNIRS25 score.

We found a significant moderate correlation between the intraoperative rNIRS65 and intraoperative rNIRS25 scores and the arterial lactate 6 hours postoperatively (*r* = 0.4, *P* < 0.0001, and *r* = 0.4, *P* = 002; respectively). The rNIRS65 and rNIRS25 scores in the first 24 hours postoperatively showed a moderate correlation to the ScvO_2_ at 2 and 24 hours after surgery (rNIRS65 score to ScvO_2_ at 2 hours: *r* = 0.6, *P* < 0.0001; rNIRS65 score to ScvO_2_ at 24 hours: *r* = 0.5, *P* < 0.0001; rNIRS25 score to ScvO_2_ at 2 hours: *r* = 0.5, *P* = 0.001; rNIRS25 score to ScvO_2_ at 24 hours: *r* = 0.4, *P* = 0.009).

In the logistic regression analysis, the only significant parameter for AKI development was the blood pressure in the first 24 hours postoperatively (*R*^2^ = 0.3, *P* = 0.02). No significance was found for age, cyanosis, bypass time or ScvO_2_ at 2 hours postoperatively.

The ROC analysis showed that, for an intraoperative rNIRS65 score cutoff value of 95 min%, the sensitivity was 96% and specificity was 40% (AUC, 0.62; *P* = 0.12) in capturing postoperative AKI. The positive predictive value (PPV) was 59%, and the negative predictive value (NPV) was 88%. For an intraoperative cutoff value for the rNIRS25 score of 23 min%, we found a sensitivity of 67% and a specificity of 76% (AUC, 0.69; *P* = 0.01). The PPV was 71%, and the NPV was 76%.

Cystatin C showed a significant increase after CPB surgery in patients with AKI starting at 24 hours postoperatively in comparison with patients without AKI (Table 5).

**Table 5:** **Pre- and postoperative cystatin C levels of infants with versus without acute kidney injury** ^**a**^

	**AKI**	**No AKI**	***P*** **-value**
Cystatin C, mg/L			
Preoperatively	1.4 (0.7 to 1.9)	1.4 (0.6 to 2.8)	0.87
Postoperatively	1.1 (0.6 to 1.3)	1.1 (0.7 to 1.2)	0.96
24 hours postoperatively	1.7 (1.0 to 2.7)	1.4 (0.8 to 2.2)	0.002
48 hours postoperatively	1.7 (1.0 to 2.1)	1.4 (0.8 to 2.6)	0.02

^a^The renal biomarker serum cystatin C is presented pre- and postoperatively and 24 and 48 hours after surgery as median and range. AKI, Acute kidney injury.

The renal biomarker NGAL in urine and plasma showed a significant increase from the preoperative to the 2 to 4 hours postoperative values (NGAL in urine: 2.4 to 25 ng/ml, *P* > 0.0001; NGAL in plasma: 35 to 41 ng/ml, *P* = 0.05). NGAL in urine or in plasma did not show a significant difference between patients with and without AKI 2 to 4 hours and 24 hours postoperatively.

## Discussion

The major finding of this study is the significant correlation of an intraoperatively measured renal NIRS score with the postoperative occurrence of AKI in infants after CPB surgery. Second, we found that renal NIRS monitoring performed better than selected biomarkers of renal injury (cystatin C, NGAL) in predicting AKI.

After CPB surgery, AKI in infants with congenital heart disease is a common complication. Its incidence is reportedly between 30% and 64% [23,24]. Occurrence of AKI leads to increased morbidity, and several authors have reported increased mortality [3,4]. Various risk factors for the development of AKI have been noted, among them age, bypass time and RACHS-1 score; however, sufficiently sensitive and specific early markers of renal injury have not been consistently defined [1]. Somatic NIRS is thought to reflect tissue perfusion. We postulated that renal rSO_2_ monitoring might have prognostic value for developing AKI. Renal NIRS could serve as a readily and continuously measurable and, most importantly, early parameter that would allow early intervention trials to modify renal outcomes.

Validated critical baselines for renal rSO_2_ are lacking and do not discern between cyanotic and acyanotic cardiac lesions. Moreover, the definition of pathological NIRS values is not consistent over studies, ranging from 50% to 80%, and do not account for the magnitude and duration of desaturations. We set the critical baseline at 65% rSO_2_ for renal NIRS according to the threshold of 45% to 55% accepted in cerebral NIRS studies [17-19] and the 10 to 20 points higher somatic than cerebral values in healthy infants [20,21]. To account for *a priori* lower NIRS values in patients with cyanotic lesions, we defined a second criterion, which includes relative decreases in regional oxygenation compared with an individually calculated baseline. We speculated that, as baseline values are already low in cyanotic patients, a parameter of a relative decrease in saturation values might be more sensitive in the detection of a perfusion compromise. In addition, the area under the threshold lines was calculated as NIRS score to account for magnitude and duration of desaturations.

In two somatic NIRS studies, it has already been shown that lower renal rSO_2_ after surgery correlates with occurrence of AKI in infants after CPB [20,25]. Only one of these studies [20] included NIRS measurements intraoperatively, but no correlation with AKI was reported. In each of these studies, different cutoff values for renal rSO_2_ were used to define critical desaturation episodes, including criteria for relevant time periods of desaturations, which impedes comparisons between studies. In contrast to these studies, we could show a significant difference in renal desaturation, by our criteria, between infants with and those without AKI developing at the earliest time point (that is, during surgery). As CPB alters oxygen delivery, this difference was most distinct after stopping CPB; however, it was consistent over 12 and 24 hours postoperatively.

Our results show that both NIRS criteria correlate with renal outcome and haemodynamic parameters and surprisingly did not differ significantly in patients with and without cyanosis, independent of the development of AKI. Efficient mechanisms seem to be functional in cyanotic patients to compensate for lower systemic arterial oxygen saturation. Therefore, we propose that, although systemic arterial oxygen saturations are lower in cyanotic patients and the majority of AKI patients were cyanotic, systemic cyanosis seems not to be the cause of the significant difference in renal NIRS values between AKI and non-AKI groups.

In various clinical studies in neonates and infants, somatic NIRS has been used as an indicator of tissue perfusion and has been established as a monitoring tool in the haemodynamic management of critically ill infants [11,13,25]. Several studies have demonstrated a moderate correlation of somatic rSO_2_ with lactate and central venous saturation as indicators of cardiac output [12,26,27]. Uncertainty remains, however, because other studies have failed to identify a correlation between regional NIRS and several cardiac output parameters [28,29]. Abdominal site NIRS [14] or a combination of cerebral and renal NIRS [27] may perform best in this regard. With more specific regard to the kidneys, a good correlation of renal vein and inferior vena cava saturation with renal NIRS in children weighing <10 kg has been demonstrated [30]. Our results support the notion that somatic NIRS is dependent on cardiac output and reflects tissue perfusion, as we could show a correlation, although it was moderate between renal NIRS scores intraoperatively and systemic lactate levels 6 and 24 hours after surgery. Further, renal NIRS scores in the first 24 hours after surgery correlated significantly with ScvO_2_ at 2 and 24 hours, as well as with systemic lactate levels at 24 hours after surgery.

In our study population, 48% of infants developed AKI after CPB surgery, which is comparable to recently published series [1,5]. Known risk factors for the development of AKI are younger age [1,3-5,31], bypass time [1-3,5], RACHS-1 score [3,5] and vasopressor use [2]. Infants, especially newborns, seem to be at highest risk for developing AKI after CPB surgery [1,5]. In our study, patients in the AKI group were significantly younger than patients in the non-AKI group, confirming these findings, whereas there was no difference in RACHS-1 scores.

CPB *per se* and haemodynamic compromise during the intra- and postoperative course could be responsible for postoperative AKI [32]. In our cohort, CPB time showed no significant difference between infants who developed AKI and infants who did not. Lactate after 24 hours in infants with AKI is significantly higher and ScvO_2_ after 24 hours in infants with AKI is significantly lower than in infants without AKI. In addition, we noted lower blood pressures and higher vasopressor requirements in the patients with AKI. Last, the number of patients with systemicopulmonary shunts was significantly higher in the AKI group. Haemodynamic conditions in systemicopulmonary shunt palliation can influence renal perfusion because of diastolic run-off and lower diastolic blood pressure [33]. In summarizing these findings, in our study population, peri- and postoperative haemodynamic compromise was a major factor in the development of postoperative AKI. Importantly, intraoperative renal NIRS reflected these pathologies at the earliest time point.

Over the last several years, renal biomarkers for use in predicting the development of AKI earlier than serum creatinine have been investigated extensively, but firm conclusions have not been reached [8]. In our present study, we confirmed earlier findings [34] that plasma cystatin C increased significantly after CPB surgery in patients with postoperative AKI, but showed a significant increase only at least 24 hours postoperatively, whereas others have noted an earlier rise [35].

With regard to serum and urinary NGAL, we did not find a significant difference between infants who developed AKI and those who did not. This finding is in contrast to several earlier reports [36-38] of impressive AUROC values, although moderate AUROC values have been noted as well [9,39]. Moreover, the optimal time point of NGAL measurements and optimal cutoff values have not been defined. We noted that in earlier studies [36-39], CPB time differed significantly between AKI and non-AKI groups, which was not the case in our study. Therefore, NGAL values may have a close correlation with CPB time and a less direct correlation with AKI incidence. In our study, neither NGAL nor cystatin C proved useful for early diagnosis of AKI after CPB. As other authors have suggested, a multimodal prognostic model that incorporates several biomarkers, clinical risk factors and NIRS values may serve best in predicting AKI [9,16].

A limitation of our study is the use of a newly defined analysis of NIRS values in infants, which has to be validated in further studies. Furthermore, we did not perform either (1) a continuous measurement of central venous saturation as an indirect real-time marker for cardiac output and tissue perfusion or (2) a thermodilution measurement as a direct real-time marker for cardiac output and tissue perfusion.

In summary, we found that continuous renal NIRS monitoring, starting intraoperatively and extending postoperatively, in infants after cardiac surgery may be a valuable and, importantly, very early parameter for predicting AKI. Of great practical value are its non-invasive and continuous character and the potential to deliver instantaneous values. We developed two methods of analysis, including absolute and relative cutoff values. rNIRS scores <65% show a high sensitivity, and relative rNIRS values <25% have acceptable specificity, both of which may be improved by refining thresholds in larger patient cohorts. In our series, intraoperative renal NIRS was superior to conventional biomarkers of renal injury for early detection of developing AKI, although a combined model that incorporates biomarkers and renal NIRS may be expected to have increased sensitivity and specificity.

## Conclusion

Renal NIRS, when performed during and after infant cardiac surgery, is a promising tool to detect early haemodynamic compromise and predict development of AKI. Renal NIRS monitoring therefore should be included in prognostic models for early identification of renal injury risk in infants during and after CPB operation and may allow the development of therapeutic strategies to avoid kidney injury during cardiac surgery in infants.

## Key message

- Renal regional NIRS, when started intraoperatively and with specific thresholds, allows very early risk detection of the development of AKI after CPB surgery in infants.

## Abbreviations

AKI: Acute kidney injury
CPB: Cardiopulmonary bypass
ICU: Intensive care unit
NGAL: Neutrophil gelatinase-associated lipocalin
NIRS: Near-infrared spectroscopy
NPV: Negative predictive value
PPV: Positive predictive value
pRIFLE: Paediatric Risk, Injury, Failure, Loss, End
RACHS-1: Risk Adjustment for Congenital Heart Surgery
rSO_2_: Regional oximetry
RRT: Renal replacement therapy
ScvO_2_: Venous oxygen saturation
